# Variability and Costs of Low-Value Preoperative Testing for Cataract Surgery Within the Veterans Health Administration

**DOI:** 10.1001/jamanetworkopen.2021.7470

**Published:** 2021-05-06

**Authors:** Seshadri C. Mudumbai, Suzann Pershing, Tom Bowe, Robin N. Kamal, Erika D. Sears, Mary T. Hawn, Dan Eisenberg, Andrea K. Finlay, Hildi Hagedorn, Alex H. S. Harris

**Affiliations:** 1 Center for Innovation to Implementation, VA Palo Alto Healthcare System, Palo Alto, California; 2 Department of Anesthesiology, Perioperative, and Pain Medicine, Stanford University School of Medicine, Stanford, California; 3 Department of Ophthalmology, Byers Eye Institute at Stanford, Stanford University School of Medicine, Stanford, California; 4 Department of Orthopedic Surgery, Stanford University School of Medicine, Stanford, California; 5 Center for Clinical Management Research, VA Ann Arbor Health Care System, Ann Arbor, Michigan; 6 Department of Surgery, Michigan Medicine, Ann Arbor; 7 Stanford–Surgical Policy Improvement Research and Education Center, Department of Surgery, Stanford University School of Medicine, Stanford, California; 8 Minneapolis Veterans Affairs Medical Center, Minneapolis, Minnesota; 9 Anesthesiology and Perioperative Care Service, VA Palo Alto Health Care System, Palo Alto, California

## Abstract

**Importance:**

The Choosing Wisely guidelines indicate that preoperative testing is often unnecessary and wasteful for patients undergoing cataract operations. However, little is known about the impact of these widely disseminated guidelines within the US Veterans Health Administration (VHA) system.

**Objective:**

To examine the extent, variability, associated factors, and costs of low-value tests (LVTs) prior to cataract operations in the VHA.

**Design, Setting, and Participants:**

This cohort study examined records of all patients receiving cataract operations within the VHA in fiscal year 2017 (October 1, 2016, to September 31, 2017). Records from 135 facilities nationwide supporting both ambulatory and inpatient surgery were included.

**Exposures:**

A laboratory test occurring within 30 days prior to cataract surgery and within 30 days after clinic evaluation.

**Main Outcomes and Measures:**

Overall national and facility-level rates and associated costs of receiving any of 8 common LVTs in the 30 days prior to cataract surgery. The patient characteristics, procedure type, and facility-level factors associated with receiving at least 1 test, the number of tests received, and receipt of a bundle of 4 tests (complete blood count, basic metabolic profile, chest radiograph, and electrocardiogram).

**Results:**

A total of 69 070 cataract procedures were identified among 50 106 patients (66 282 [96.0%] men; mean [SD] age, 71.7 [8.1] years; 53 837 [77.9%] White, 10 292 [14.9%] Black). Most of the patient population had either overweight (23 292 [33.7%] patients) or obesity (27 799 [40.2%] patients). Approximately 49% of surgical procedures (33 424 procedures) were preceded by 1 or more LVT with an overall LVT cost of $2 597 623. Among patients receiving LVTs, electrocardiography (7434 patients [29.9%]) was the most common, with some patients also receiving more costly tests, including chest radiographs (489 patients [8.2%]) and pulmonary function tests (127 patients [3.4%]). For receipt of any LVT, the intraclass correlation coefficient was 0.61 (*P* < .001) at the facility level and 0.06 (*P* < .001) at the surgeon level, indicating the substantial contribution of the facility to amount of tests given.

**Conclusions and Relevance:**

Despite existing guidelines, use of LVTs prior to cataract surgery is both common and costly within a large, national integrated health care system. Our results suggest that publishing evidence-based guidelines alone—such as the Choosing Wisely campaign—may not sufficiently influence individual physician behavior, and that system-level efforts to directly deimplement LVTs may therefore necessary to effect sustained change.

## Key Points

**Question:**

To what extent are low-value preoperative tests used before cataract surgery in the US Veterans Health Administration, and what is the variability and cost of these tests?

**Findings:**

In this cohort study including 69 070 cataract procedures performed among 50 106 patients, almost half were preceded by at least 1 low-value test. Compared with low-complexity facilities, higher facility-level complexity was associated with higher odds of receiving a 4-test bundle.

**Meaning:**

Despite the dissemination of Choosing Wisely guidelines surrounding cataract surgery within a large, integrated health care system, these results suggest that low-value tests continue to be widespread, and that more intensive deimplementation approaches are required.

## Teaser

This cohort study of Veterans Health Administration records examines the prevalence of low-value preoperative tests for eye cataract operations and associations with patient demographic characteristics, procedure type, and facility size.

## Introduction

Advances in technology starting in the 1960s led to the introduction of a multiphasic battery of preoperative laboratory tests.^1^ Over time, because of the assumption that early and frequent testing could be used for presymptomatic diagnosis and optimization, screening testing became widespread throughout the US. However, high rates of false positives were observed, which resulted in “million dollar workups” that substantially contributed to increases in overall health care costs.^2,3,4^

In response to this trend, the Choosing Wisely campaign was initiated by the American Board of Internal Medicine Foundation in February 2013, seeking to advance a national dialogue on avoiding unnecessary medical tests, treatments, and procedures.^5^ This initiative has been endorsed by the American Academy of Ophthalmology and American Society of Anesthesiologists, particularly around preoperative testing—low-value tests (LVTs)—and is included in a board-recertification module for the American Board of Ophthalmology.^6^ Recent Cochrane guidelines suggest that for patients undergoing low-risk surgery, baseline laboratory studies such as complete blood count, basic or comprehensive metabolic panels, and coagulation studies often are unnecessary.^7^ Clinicians are not likely to change their surgical plan or delay surgery based on these tests.^8^ In addition, preoperative tests, such as electrocardiography and cardiac stress tests, have not been found to decrease adverse events nor improve outcomes for low-risk procedures, such as cataract operations.^1,9^

Cataract operations are the most common elective surgical procedures among Medicare beneficiaries and older populations, with 1.7 million procedures performed annually. Data among this population indicate that preoperative testing was often found prior to cataract operations and had robust associations with clinician practice patterns.^10^ As part of ongoing quality of care evaluations, it is often necessary to evaluate the extent to which real-world preoperative testing continues to conform to guidelines, as well as to assess the relative contribution of patient and facility-level factors.

The number of cataract surgeries is expected to increase over the next decade within the Veterans Health Administration (VHA). Serving primarily an older, male population, the VHA conducts surgical procedures across 150 major facilities throughout the US. With a common electronic health record and detailed longitudinal follow-up, a unique opportunity exists to evaluate post–Choosing Wisely use of LVTs within an integrated health care system and examine factors associated with greater utilization of LVTs.^11^ Therefore, as part of a quality of care study, our goals were to: (1) determine the overall and facility-level proportion of patients receiving any of 8 common preoperative LVTs in the 30 days prior to a cataract operation; (2) examine factors at the level of patient, surgeon, and facility that are associated with receiving at least 1 LVT, the amount of tests received, and receiving a bundle of 4 common preoperative LVTs (complete blood count, basic metabolic profile, chest radiograph, and electrocardiogram); and (3) estimate overall costs of LVTs in fiscal year (FY) 2017.

## Methods

### Data Source and Cohort Definition

We received approval and a waiver of informed consent by the Stanford University institutional review board because the study used existing electronic health record data. We extracted records from the VA Corporate Data Warehouse for FY 2017 (October 1, 2016, to September 30, 2017) for a cohort study of patients who underwent cataract surgery using *Current Procedural Terminology* (*CPT*) codes (66982, 66983, and 66984) regardless of American Society of Anesthesiologists Physical Status (ASA-PS) classifications. Since patients often get the other eye operated on for cataract surgery at a later date, we considered the subsequent procedure as a unique procedure and included it for analysis. Within the VHA, it is common for cataract procedures to be conducted within an operating room setting, usually under monitored anesthetic care (MAC) in lieu of general anesthesia (GA). This report followed the Strengthening the Reporting of Observational Studies in Epidemiology (STROBE) reporting guideline for cohort studies.

### Preoperative Tests

We used methods developed for a prior study in carpal tunnel surgery patients within the VHA to identify preoperative LVTs using *CPT* codes.^12^ A test was considered preoperative if it occurred within 30 days prior to cataract surgery and if it occurred within 30 days after an encounter in a specific VHA clinic where preoperative screening tests are typically ordered, including those that are explicitly dedicated to preoperative care (eg, VHA clinic “stop” code 416–Pre-Surgery Evaluation by Non-MD; 419–Anesthesia Pre-Op/Post-Op Consult; 432–Pre-Surgery Evaluation by MD; 433–Pre-Surgery Evaluation by Nursing) or are likely related to preoperative care (ie, VHA clinic “stop” code 407–Ophthalmology). We excluded tests occurring within 30 days of noncataract procedures (eg, coronary artery bypass graft) that might have justified testing. We determined this list a priori (eTable 1 in the Supplement). Tests included basic metabolic panel (BMP); complete blood count (CBC); cardiac stress tests; urinalysis; chest radiograph; pulmonary function testing; electrocardiograms (ECG); and transthoracic echocardiograms. We were also interested in receipt of a “legacy bundle” of tests (ECG, radiograph, CBC, and BMP).

### Other Patient and Facility Characteristics

To help us account for other variables that might affect the receipt of a LVT, we collected information on the following: sociodemographic characteristics (ie, age, gender, race/ethnicity [American Indian, Asian, Black, Hawaiian, and White], and marital status) and body mass index (calculated as weight in kilograms divided by height in meters squared). We then identified comorbidities, like congestive heart failure, as described by Elixhauser.^13^ The VHA hosts an extensively curated data warehouse that tracks comorbidities closely and accurately.^14^ We also collected requested anesthesia type (GA vs MAC). Finally, we identified characteristics of facilities where cataract operations were performed, including facility surgical complexity (ambulatory basic, ambulatory advanced, inpatient standard, inpatient intermediate, inpatient complex) and annual cataract operation volume.

### Statistical Analysis

We first determined overall and facility-level proportions of cataract surgical procedures that were preceded by any preoperative test. Among patients who received at least 1 test, we tabulated the number and relative frequency of each type of preoperative test and associated costs. We assigned each patient a value equaling the total number of Elixhauser comorbidities.^13,15^

We then examined associations between patient-level characteristics (eg, demographic information), procedural characteristics (eg, anesthesia type), and facility-level characteristics (eg, facility surgical complexity) with receipt of any test, number of tests, and receipt of a legacy bundle. We prescreened variables in bivariate regressions at *P* < .25 before including them in the multivariable regression (eTable 2 in the Supplement). Within hierarchical mixed models, we clustered by facility, surgeon, and patient: data on procedures were nested within patients (for patients with >1 operation), which were nested within surgeons, which were nested within facility. Surgical procedures with missing data were excluded. We used mixed-effects logistic regression for analysis of receipt of any test or receiving a legacy bundle. To analyze the number of tests, we used quasi-Poisson regression with negative binomial distribution accounting for overdispersion. Since a second eye cataract may potentially affect results, we conducted a sensitivity analysis limited to patients with their first operation, evaluating the receipt of any LVT. Odds ratios, 95% confidence intervals, and *P* values were produced for all regression model coefficients. We then calculated intraclass correlation coefficients (ICCs) to quantify the proportion of variance at the patient-, surgeon-, and facility-level. Wu et al^16^ have described how standard error measures used for generalized linear mixed models may inaccurately report variance, and so we present ICCs as point estimates without additional measures of variance. Centers for Medicare & Medicaid Services (CMS) reimbursement fee schedules were used as a reference for physician and facility fees, and the CMS clinical laboratory fee schedule for 2017 was used to assign a cost to each test, which we then added to determine overall and facility-level costs. The total cost per CPT code is presented in eTable 3 in the Supplement. All hypothesis tests were 2-sided with *P* value <.05 for statistical significance. All statistical analyses were conducted using R version 4.0.1 (R Foundation for Statistical Computing).

## Results

Characteristics of patients, procedures, and facilities are provided in Table 1 and Table 2. For FY 2017, there was a total of 69 070 cataract operations among 50 106 patients (31 444 patients had 1 operation, 18 423 patients had 2 operations, and 202 had 3 operations or more). Overall, our patient population had a mean (SD) age of 71.7 (8.1) years; most patients were men (66 282 [96.0%]), White (53 837 [77.9%]), married (37 529 [54.3%]), either overweight (23 292 [33.7%]) or had obesity (27 799 [40.2%]), and had 3 or more Elixhauser comorbidities (45 308 [65.6%]). Nearly all patients (67 973 [98.4%]) received MAC for their surgery. Approximately 84% of surgical procedures were conducted at a facility supporting either intermediate (12 375 [17.9%] procedures) or complex (45 522 [65.9%] procedures) inpatient surgery. Overall, mean (SD) annual cataract surgery volumes were 729 (328) cases per facility.

**Table 1.** Patient and Procedure Characteristics of Veterans Health Administration Cataract Surgical Procedures in Fiscal Year 2017

Characteristics	Patients, No. (%)		
	Received any preoperation test		Total (n = 69 070)
	No (n = 35 646)	Yes (n = 33 424)	
Age, mean (SD), y	71.6 (8.0)	71.7 (8.1)	71.7 (8.1)
Gender			
Male	34 133 (95.8)	32 149 (96.2)	66 282 (96.0)
Female	1513 (4.2)	1275 (3.8)	2788 (4.0)
Race/ethnicity			
American Indian	410 (1.2)	280 (0.8)	690 (1.0)
Asian	236 (0.7)	175 (0.5)	411 (0.6)
Black	5024 (14.1)	5268 (15.8)	10 292 (14.9)
Hawaiian	329 (0.9)	268 (0.8)	597 (0.9)
White	27 947 (78.4)	25 890 (77.5)	53 837 (77.9)
Missing	1700 (4.8)	1543 (4.6)	3259 (4.7)
Marital status			
Single	1042 (2.9)	1092 (3.3)	2134 (3.1)
Married	19 414 (54.5)	18 115 (54.2)	37 529 (54.3)
Divorced	9574 (26.9)	8770 (26.2)	18 344 (26.6)
Widowed	2838 (8.0)	2647 (7.9)	5485 (7.8)
Never married	2635 (7.4)	2698 (8.1)	5333 (7.6)
Missing	143 (0.4)	102 (0.3)	245 (0.4)
BMI			
Normal (healthy weight)	6391 (17.9)	6115 (18.3)	12 506 (18.1)
Low	416 (1.2)	423 (1.3)	839 (1.2)
Obesity	14 233 (39.9)	13 576 (40.6)	27 799 (40.2)
Overweight	12 024 (33.7)	11 268 (33.7)	23 292 (33.7)
Missing	2592 (7.3)	2042 (6.1)	4634 (6.7)
Elixhauser comorbidities, No.^a^			
0	2311 (6.5)	1631 (4.9)	3942 (5.7)
1	4867 (13.7)	3711 (11.1)	8578 (12.4)
2	6028 (16.9)	5214 (15.6)	11 242 (16.3)
≥3	22 440 (63.0)	22 868 (68.4)	45 308 (65.6)
Procedure characteristics			
General anesthesia	450 (1.3)	647 (1.9)	1097 (1.6)
Monitored anesthesia care	35 196 (98.7)	32 777 (98.1)	67 973 (98.4)

Abbreviation: BMI, body mass index (calculated as weight in kilograms divided by height in meters squared).

^a^ Elixhauser comorbidities described in Yurkovich et al^13^ and Thompson et al.^15^

**Table 2.** Facility Characteristics of Veterans Health Administration Cataract Surgical Procedures in Fiscal Year 2017^a^

Facility characteristics	Patients, No. (%)^a^			
	Received any preoperation test			Total (n = 69 070)
	No (n = 35 646)		Yes (n = 33 424)	
Facility surgical complexity				
Ambulatory				
Basic	1301 (3.6)	1880 (5.6)		3181 (4.6)
Advanced	1076 (3.0)	2337 (7.0)		3413 (4.9)
Inpatient				
Standard	2337 (6.6)	803 (2.4)		3140 (4.5)
Intermediate	6381 (17.9)	5994 (17.9)		12 375 (17.9)
Complex	23 900 (67.0)	21 622 (64.7)		45 522 (65.9)
Unknown	651 (1.8)	788 (2.4)		1439 (2.1)
Facilities annual cataract surgery volume, mean (SD)	738 (323)	719 (332)		729 (328)

^a^ 135 total facilities analyzed.

### Rates and Costs of LVT

Approximately 49% (33 424) of cataract operations were preceded by at least 1 preoperative LVT (Table 3). The overall cost of LVTs across all facilities in FY 2017 was $2 597 623. Of the 49% of cataract operations for which the patient received at least 1 LVT, the number of tests obtained ranged from 1 to 8. For all patients, the median (interquartile range [IQR]) was 0 (0-2) for number of tests obtained. While the 3 most common types of tests had lower costs (ECG, 7434 [29.9%]; CBC, 6231 [28.2%]; and BMP, 4145 [19.1%]), we nevertheless noted that 13.1% of patients were also receiving more costly tests, including chest radiographs and pulmonary function tests. The Figure demonstrates the percentage of cataract procedures receiving at least 1 LVT at VHA facilities in FY 2017. There were 12 facilities in which over 75% of cataract surgery patients received at least 1 LVT. Over 50% of facilities had absolute totals of LVT greater than 600 during the study period. We also found that approximately 33% of LVTs were in facilities without designated preoperative assessment clinics (eg, anesthesiology screening).

**Table 3.** Low-Value Preoperative Tests Received by Veterans Health Administration Patients for Cataract Surgical Procedures in Fiscal Year 2017

Preoperative test	Patients receiving ≥1 tests, No. (%)	Total tests, No.	Total cost, $	Cost per test, $
Complete blood count	6231 (28.2)	22 095	228 515	10.34
Basic metabolic profile	4145 (19.1)	21 704	271 875	12.50
Electrocardiography	7434 (29.9)	24 862	352 888	14.19
Urinalysis	795 (9.6)	8282	30 359	3.66
Chest radiograph	489 (8.2)	5969	608 826	101.99
Pulmonary function tests	127 (3.4)	3747	70 476	18.80
Transthoracic echocardiogram	8 (1.0)	840	542 811	646.20
Cardiac stress test	35 (0.5)	693	491 874	709.77
Total	43 302 (49.1)^a^	88 193	2 597 623	29.45

^a^ Percentage of surgical procedures getting at least 1 test.

**Figure.**
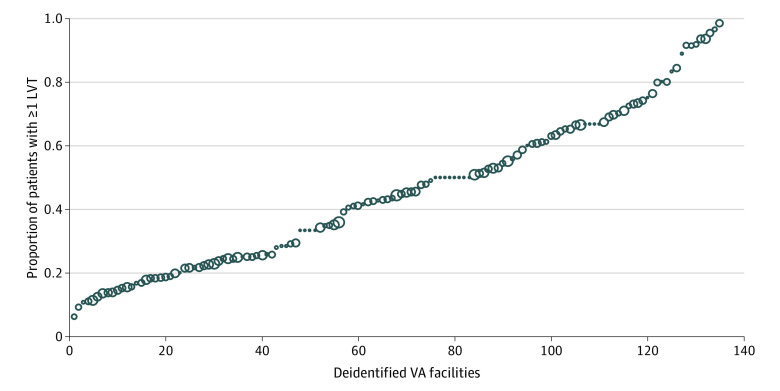
Variation in Low-Value Testing Prior to Cataract Surgical Procedures in 135 Veterans Health Administration Facilities Each dot indicates 1 facility with size proportional to the number of cataract procedures at the facility.

### Factors Associated With Receipt of LVTs

Table 4 presents results of the logistic regression examining associations between patient, procedure, and facility characteristics and receipt of at least 1 test. The strongest factors for receipt of any test included being Black, having a greater number of comorbidities, and the receipt of general anesthesia. The ICCs were 0.89 (*P* < .001) at the VHA facility level, 0.51 (*P* < .001) at the surgeon level, and 0.50 (*P* = .001) at the patient level, indicating the substantial contribution of the facility to testing. For our sensitivity analysis (first eye surgery only), the ICCs were 0.65 (*P* < .001) at the VHA facility level and 0.16 (*P* < .04) at the surgeon level (eTable 4 in the Supplement). eTables 5 and 6 in the Supplement provide the results of our multivariable regression models to evaluate the role of various factors associated with receipt of the legacy bundle and the number of tests. When we evaluated receipt of a bundle by facility type, inpatient standard facilities were associated with low odds for receipt of a 4-test legacy bundle compared with low-complexity facilities (OR, 0; 95% CI, 0-0; *P* < .001). The ICC at the facility level for receipt of a bundle was 0.98 (*P* < .001).

**Table 4.** Mixed-Effects Logistic Regression Model Describing the Association Between Patient, Surgery, and Facility Characteristics and Receipt of Any Preoperative Test Before Cataract Surgery in Fiscal Year 2017^a^

Characteristics	Total operations, No.	OR (95% CI)	*P* value
Facility surgical complexity			
Ambulatory			
Advanced	3413	1 [Reference]	[Reference]
Basic	3181	0.31 (0.04-2.54)	.28
Inpatient			
Standard	3140	0.28 (0.05-1.48)	.13
Intermediate	12 375	0.27 (0.04-1.63)	.15
Complex	45 522	0.03 (0.00-0.25)	<.001
Unknown	1439	0.28 (0.02-2.91)	.29
Annual facility cataract operations volume	NA	0.99 (0.99-1.00)	.16
Age (per 1 y)	NA	0.99 (0.99-1.00)	.66
Women (vs men)	2788	1.08 (0.92-1.27)	>.99
Race/ethnicity			
Non-Hispanic White	53 837	1 [Reference]	[Reference]
Asian	411	0.91 (0.62-1.34)	.64
Black or African American	10 292	1.16 (1.06-1.27)	<.001
Hawaiian	597	0.79 (0.56-1.10)	.16
American Indian	690	0.99 (0.99-1.14)	.16
Marital status			
Married	37 529	1 [Reference]	[Reference]
Single	2134	1.07 (0.90-1.28)	.41
Divorced	18 344	0.98 (0.81-1.17)	.83
Widowed	5485	0.96 (0.78-1.18)	.70
Never married	5333	1.04 (0.85-1.28)	.64
BMI			
Normal (healthy weight)	12 506	1 [Reference]	[Reference]
Low	839	0.74 (0.56-0.97)	.03
Obesity	27 799	0.99 (0.91-1.07)	.82
Overweight	23 292	1.02 (0.93-1.11)	.62
Elixhauser index (per No. of comorbidities)	NA	1.13 (1.12-1.14)	<.001
Surgery characteristics			
General anesthesia	1097	1 [Reference]	[Reference]
Monitored anesthesia care	67 973	0.66 (0.53-0.83)	<.001

Abbreviations: BMI, body mass index (calculated as weight in kilograms divided by height in meters squared); OR, odds ratio.

^a^ Analysis conducted at a procedure level (69 070 total surgical procedures).

## Discussion

About half of patients presenting for cataract surgical procedures within an integrated health care system, the VHA, received at least 1 preoperative LVT in FY 2017—approximately 4 years after release of the Choosing Wisely campaign by the American Academy of Ophthalmology. Our findings demonstrate patterns of low-value testing across a large sample (69 070 surgical procedures) in a nationwide, geographically diverse network of hospitals for multiple important characteristics: facility type (ranging from those supporting basic ambulatory procedures to others that routinely conduct complex inpatient surgical procedures); receipt of MAC or GA; patient gender, age, and race/ethnicity; and various comorbidities. While the most common LVT was an ECG, we noted frequent use of lower-cost blood tests as well as more expensive chest radiographs and cardiac stress tests.

Our findings are pertinent to clinical practice given the well-established guidelines by the Choosing Wisely Campaign for limiting preoperative testing before low-risk surgical procedures.^1,5^ Systemic complications following cataract surgery are extremely rare, with 30-day mortality being below 0.1%.^1^ However, over the past decade, data indicate that cataract procedures have a substantial likelihood for patients to be seen for a preoperative consult, many of which include routine testing.^17^ As the US population continues to age, these evaluations are likely to grow owing to the existence of multiple comorbidities in older cataract surgery patients compared with the typical surgical patient.^1,18^ Although preoperative testing can potentially be used for stratification and reduction of risk for major procedures, in the setting of low-risk operations its value has not been demonstrated.^19,20^ Routine preoperative medical testing did not change the risk of intraoperative or postoperative ocular adverse events when compared with selective or no testing in 3 large randomized clinical trials of cataract surgery patients.^1^ Most events were cardiovascular and occurred during the intraoperative period, further suggesting that routine preoperative testing does not increase the safety of cataract operations.

Our novel models incorporated a variety of factors, such as facility-level complexity that may indicate systemic practices as well as annual cataract case volumes, type of anesthesia, and detailed comorbidities. Our rates of LVT (49%) were comparable with those found in pre-Choosing Wisely Medicare cohorts (53%). We also noted the role of facility complexity in receipt of a 4-test preoperative testing bundle. Even more so than comorbidities, nonpatient factors such as location of surgery are strongly associated with preoperative laboratory testing in aggregate.^19^ Other population-level studies have previously evaluated rates and patterns of preoperative testing in US (ie, Medicare) and Canadian cohorts before the Choosing Wisely campaign, and found elevated levels of low-value testing.^10,19^ Our findings are striking in that frequent low-value testing persists approximately 4 years after the initiation of the Choosing Wisely campaign, and that high levels of low-value testing are present in a health care system without financial incentives for ordering tests (VHA clinicians are salary-based, not compensated in a fee-for-service model). Our approach used CMS costs, which allowed us to capture the estimated societal burden of LVTs. These results demonstrate that publishing evidence-based guidelines or recommendations such as Choosing Wisely alone does not necessarily change individual physician behavior.^21,22^

A principal aim of our study therefore was to evaluate the variance across the 135 VHA facilities that conduct cataract surgical procedures. The fact that some facilities are testing close to 100% of their patients while others are closer to 0% suggests the need to evaluate factors at the level of patient, health professional, and hospital that are associated with more low-value care. Furthermore, by evaluating the intraclass correlation coefficients within each hierarchical level, we can determine the relative importance of that level as well as individual factor contributions. These data thereby suggest opportunities for and inform strategies for deimplementation.

Our results have several immediate implications for preoperative testing. First, rather than solely focusing on LVTs within the context of a standard preoperative clinic, additional efforts should be directed toward identifying alternative, nontraditional approaches to minimize LVT requests while retaining advantages associated with a clinic setting.^20,23^ In our study, we noted that about 33% of LVTs were in facilities that typically do not have a designated preoperative clinic. One suggested approach is to use directed testing at the time of initial evaluation by the surgeon guided by simple online risk stratification tools and early objective physiological assessments.^24^ While this study evaluated VHA settings without a preoperative clinic as part of a qualitative deimplementation study, several principles for the benefits of preoperative clinics nevertheless need to be kept in mind. These benefits include selective testing based on risk assessments after a detailed clinical history and physical examination, rather than age-based and bundled ordering of tests.^1^ In addition, alternative approaches should help to avoid last-minute operating room delays and potential cancellations that often drive testing.

Second, system-level interventions beyond guideline dissemination are necessary to change clinician behavior for preoperative testing.^25^ As efforts are underway to achieve lower rates of low-value testing and costly medical practices, our results support roles for health care systems and physicians to (1) enable interventions at a systems level for the goals outlined in the Choosing Wisely guidelines; and (2) deploy deimplementation efforts to change clinician behavior and make it easier to achieve these goals. With current fee-for-service regulations, health care systems can still be paid for tests regardless of their need. However, new requirements from the CMS could require external reporting of Choosing Wisely metrics, such as rates and types of preoperative testing in cataract surgery.^26,27^ Efforts can occur at the level of a hospital or facility in parallel that employ approaches developed from the field of implementation science to instead deimplement or change clinician behavior. In addition, studying how test results factor into a clinician’s decision making (like the decision to proceed) could help in designing education efforts.

Finally, it is possible that practice patterns within the VHA may reflect community standards outside of VHA, which are informed by financial drivers such as Medicare reimbursement rates. Future efforts such as episode-based cost measures under the merit-based incentive payment system arm of the Medicare Access and Children’s Health Insurance Program Reauthorization Act quality payment program may influence and help to reduce use of unnecessary services such as preoperative LVTs.

### Limitations

This study has several limitations. First, while we developed rigorous methods to establish that tests were preoperative and had been validated previously, it is uncertain whether all tests were ordered as a result of the planned surgery.^12^ Second, to avoid including just subpopulations where testing may have been justified, we selected all patients who underwent cataract surgical procedures within 1 fiscal year and conducted a broad, epidemiologic, population-level study and adjusted for a diverse set of predictive factors including detailed comorbidities. Third, VHA is a capitated, publicly funded health care system, so patterns and drivers of preoperative test ordering behavior may be different than other contexts, including non-VHA health care systems. Fourth, second eye cataract operations performed after a given patient’s first eye operation may have lower rates of preoperative LVT if the procedures were performed in close succession and LVTs applied for both operations. Fifth, we used anesthesia technique as requested by the surgeon at time of scheduling, rather than the anesthesia technique applied, for which data are not readily available. Our results deserve further study in non-VHA cohorts, which may be subject to different financial drivers and facility requirements.

## Conclusions

In summary, preoperative LVTs for patients undergoing cataract surgery appear to be common in the VHA 4 years after release of the Choosing Wisely campaign. We noted this across all types of facilities and patients. However, the contribution of facility-level complexity to receipt of LVTs requires further clarification. Our results also highlight the need to qualitatively study sites that have successfully improved in the hope of identifying feasible deimplementation strategies.^28^

## Notes

Supplement. **eTable 1.** Noncataract Procedures That Were Considered to Have Justified Testing **eTable 2.** Bivariate Analysis Describing the Association Between Patient, Surgery and Facility Characteristics, and Receipt of Any Preoperative Test Before Cataract Surgery in Fiscal Year 2017 **eTable 3.** Costs Per CPT Code for Laboratory Tests **eTable 4.** Sensitivity Analysis: Mixed-Effects Logistic Regression Model for First Eye Surgery Describing the Association Between Patient, Surgery and Facility Characteristics, and Receipt of Any Preoperative Test Before Cataract Surgery in Fiscal Year 2017 **eTable 5.** Mixed-Effects Logistic Regression Model Describing the Association Between Patient, Surgery and Facility Characteristics, and Receipt of Preoperative Test Bundle Before Cataract Surgery in Fiscal Year 2017 **eTable 6.** Mixed-Effects Poisson Regression Model Describing the Association Between Patient, Surgery and Facility Characteristics, and Number of Preoperative Tests Before Cataract Surgery in Fiscal Year 2017
