# Supplementary material for: Variability and Costs of Low-Value Preoperative Testing for Cataract Surgery Within the Veterans Health Administration
Source: JAMA Netw Open. 2021 May 6;4(5):e217470. doi: 10.1001/jamanetworkopen.2021.7470 (PMC8103225; doi:10.1001/jamanetworkopen.2021.7470)
Supplement: Supplement. — eTable 1. Noncataract Procedures That Were Considered to Have Justified Testing eTable 2. Bivariate Analysis Describing the Association Between Patient, Surgery and Facility Characteristics, and Receipt of Any Preoperative Test Before Cataract Surgery in Fiscal Year 2017 eTable 3. Costs Per CPT Code for Laboratory Tests eTable 4. Sensitivity Analysis: Mixed-Effects Logistic Regression Model for First Eye Surgery Describing the Association Between Patient, Surgery and Facility Characteristics, and Receipt of Any Preoperative Test Before Cataract Surgery in Fiscal Year 2017 eTable 5. Mixed-Effects Logistic Regression Model Describing the Association Between Patient, Surgery and Facility Characteristics, and Receipt of Preoperative Test Bundle Before Cataract Surgery in Fiscal Year 2017 eTable 6. Mixed-Effects Poisson Regression Model Describing the Association Between Patient, Surgery and Facility Characteristics, and Number of Preoperative Tests Before Cataract Surgery in Fiscal Year 2017 [file jamanetwopen-e217470-s001.pdf]

Supplemental Online Content

Mudumbai SC, Pershing S, Bowe T, et al. Variability and costs of low-value preoperative testing for cataract surgery within the Veterans Health Administration. *JAMA Netw Open*. 2021;4(5):e217470. doi:10.1001/jamanetworkopen.2021.7470

eTable 1. Noncataract Procedures That Were Considered to Have Justified Testing
eTable 2. Bivariate Analysis Describing the Association Between Patient, Surgery and Facility Characteristics, and Receipt of Any Preoperative Test Before Cataract Surgery in Fiscal Year 2017
eTable 3. Costs Per CPT Code for Laboratory Tests
eTable 4. Sensitivity Analysis: Mixed-Effects Logistic Regression Model for First Eye Surgery Describing the Association Between Patient, Surgery and Facility Characteristics, and Receipt of Any Preoperative Test Before Cataract Surgery in Fiscal Year 2017
eTable 5. Mixed-Effects Logistic Regression Model Describing the Association Between Patient, Surgery and Facility Characteristics, and Receipt of Preoperative Test Bundle Before Cataract Surgery in Fiscal Year 2017
eTable 6. Mixed-Effects Poisson Regression Model Describing the Association Between Patient, Surgery and Facility Characteristics, and Number of Preoperative Tests Before Cataract Surgery in Fiscal Year 2017

This supplemental material has been provided by the authors to give readers additional information about their work.

© 2021 Mudumbai SC et al. *JAMA Network Open.*

eTable 1. **Noncataract procedures that were considered to have justified testing.** CPT=Current Procedural Terminology		
**CPT Code**	**CPT Name**	**CPT Description**
10120	REMOVE FOREIGN BODY	INCISION AND REMOVAL OF FOREIGN BODY, SUBCUTANEOUS TISSUES; SIMPLE
11042	DEB SUBQ TISSUE 20 SQ CM/<	DEBRIDEMENT, SUBCUTANEOUS TISSUE (INCLUDES EPIDERMIS AND DERMIS, IF PERFORMED); FIRST 20 SQ CM OR LESS
12042	INTMD RPR N-HF/GENIT2.6-7.5	REPAIR, INTERMEDIATE, WOUNDS OF NECK, HANDS, FEET AND/OR EXTERNAL GENITALIA; 2.6 CM TO 7.5 CM
12051	INTMD RPR FACE/MM 2.5 CM/<	REPAIR, INTERMEDIATE, WOUNDS OF FACE, EARS, EYELIDS, NOSE, LIPS AND/OR MUCOUS MEMBRANES; 2.5 CM OR LESS
14040	TIS TRNFR F/C/C/M/N/A/G/H/F	ADJACENT TISSUE TRANSFER OR REARRANGEMENT, FOREHEAD, CHEEKS, CHIN, MOUTH, NECK, AXILLAE, GENITALIA, HANDS AND/OR FEET; DEFECT 10 SQ CM OR LESS
20680	REMOVAL OF SUPPORT IMPLANT	REMOVAL OF IMPLANT; DEEP (EG, BURIED WIRE, PIN, SCREW, METAL BAND, NAIL, ROD OR PLATE)
27187	REINFORCE HIP BONES	PROPHYLACTIC TREATMENT (NAILING, PINNING, PLATING OR WIRING) WITH OR WITHOUT METHYLMETHACRYLATE, FEMORAL NECK AND PROXIMAL FEMUR
29580	APPLICATION OF PASTE BOOT	STRAPPING; UNNA BOOT
29822	SHO ART HRS SRG LMTD DBRDMT	ARTHROSCOPY, SHOULDER, SURGICAL; DEBRIDEMENT, LIMITED, 1 OR 2 DISCRETE STRUCTURES (EG, HUMERAL BONE, HUMERAL ARTICULAR CARTILAGE, GLENOID BONE, GLENOID ARTICULAR CARTILAGE, BICEPS TENDON, BICEPS ANCHOR COMPLEX, LABRUM, ARTICULAR CAPSULE, ARTICULAR SIDE OF THE ROTATOR CUFF, BURSAL SIDE OF THE ROTATOR CUFF, SUBACROMIAL BURSA, FOREIGN BODY[IES])
29824	SHO ARTHRS SRG DSTL CLAVICLC	ARTHROSCOPY, SHOULDER, SURGICAL; DISTAL CLAVICULECTOMY INCLUDING DISTAL ARTICULAR SURFACE (MUMFORD PROCEDURE)
29826	SHO ARTHRS SRG DECOMPRESSION	ARTHROSCOPY, SHOULDER, SURGICAL; DECOMPRESSION OF SUBACROMIAL SPACE WITH PARTIAL ACROMIOPLASTY, WITH CORACOACROMIAL LIGAMENT (IE, ARCH) RELEASE, WHEN PERFORMED (LIST SEPARATELY IN ADDITION TO CODE FOR PRIMARY PROCEDURE)
30140	RESECT INFERIOR TURBINATE	SUBMUCOUS RESECTION INFERIOR TURBINATE, PARTIAL OR COMPLETE, ANY METHOD
30520	REPAIR OF NASAL SEPTUM	SEPTOPLASTY OR SUBMUCOUS RESECTION, WITH OR WITHOUT CARTILAGE SCORING, CONTOURING OR REPLACEMENT WITH GRAFT
30901	CONTROL OF NOSEBLEED	CONTROL NASAL HEMORRHAGE, ANTERIOR, SIMPLE (LIMITED CAUTERY AND/OR PACKING) ANY METHOD
31231	NASAL ENDOSCOPY DX	NASAL ENDOSCOPY, DIAGNOSTIC, UNILATERAL OR BILATERAL (SEPARATE PROCEDURE)
31233	NSL/SINS NDSC DX MAX SINUSC	NASAL/SINUS ENDOSCOPY, DIAGNOSTIC; WITH MAXILLARY SINUSOSCOPY (VIA INFERIOR MEATUS OR CANINE FOSSA PUNCTURE)
31267	ENDOSCOPY MAXILLARY SINUS	NASAL/SINUS ENDOSCOPY, SURGICAL, WITH MAXILLARY ANTROSTOMY; WITH REMOVAL OF TISSUE FROM MAXILLARY SINUS
31535	LARYNGOSCOPY W/BIOPSY	LARYNGOSCOPY, DIRECT, OPERATIVE, WITH BIOPSY;
31575	DIAGNOSTIC LARYNGOSCOPY	LARYNGOSCOPY, FLEXIBLE; DIAGNOSTIC
31622	DX BRONCHOSCOPE/WASH	BRONCHOSCOPY, RIGID OR FLEXIBLE, INCLUDING FLUOROSCOPIC GUIDANCE, WHEN PERFORMED; DIAGNOSTIC, WITH CELL WASHING, WHEN PERFORMED (SEPARATE PROCEDURE)
31623	DX BRONCHOSCOPE/BRUSH	BRONCHOSCOPY, RIGID OR FLEXIBLE, INCLUDING FLUOROSCOPIC GUIDANCE, WHEN PERFORMED; WITH BRUSHING OR PROTECTED BRUSHINGS
31628	BRONCHOSCOPY/LUNG BX EACH	BRONCHOSCOPY, RIGID OR FLEXIBLE, INCLUDING FLUOROSCOPIC GUIDANCE, WHEN PERFORMED; WITH TRANSBRONCHIAL LUNG BIOPSY(S), SINGLE LOBE

© 2021 Mudumbai SC et al. *JAMA Network Open.*

31652	BRONCH EBUS SAMPLNG 1/2 NODE	BRONCHOSCOPY, RIGID OR FLEXIBLE, INCLUDING FLUOROSCOPIC GUIDANCE, WHEN PERFORMED; WITH ENDOBRONCHIAL ULTRASOUND (EBUS) GUIDED T RANSTRACHEAL AND/OR TRANSBRONCHIAL SAMPLING (EG, ASPIRATION[S]/BIOPSY[IES]), ONE OR TWO MEDIASTINAL AND/OR HILAR LYMPH NODE STATIONS OR STRUCTURES
32554	ASPIRATE PLEURA W/O IMAGING	THORACENTESIS, NEEDLE OR CATHETER, ASPIRATION OF THE PLEURAL SPACE; WITHOUT IMAGING GUIDANCE
33213	INSERT PULSE GEN DUAL LEADS	INSERTION OF PACEMAKER PULSE GENERATOR ONLY; WITH EXISTING DUAL LEADS
33216	INSERT 1 ELECTRODE PM-DEFIB	INSERTION OF A SINGLE TRANSVENOUS ELECTRODE, PERMANENT PACEMAKER OR IMPLANTABLE DEFIBRILLATOR
35301	RECHANNELING OF ARTERY	THROMBOENDARTERECTOMY, INCLUDING PATCH GRAFT, IF PERFORMED; CAROTID, VERTEBRAL, SUBCLAVIAN, BY NECK INCISION
36147	ACCESS AV DIAL GRFT FOR EVAL	INTRODUCTION OF NEEDLE AND/OR CATHETER, ARTERIOVENOUS SHUNT CREATED FOR DIALYSIS (GRAFT/FISTULA); INITIAL ACCESS WITH COMPLETE RADIOLOGICAL EVALUATION OF DIALYSIS ACCESS, INCLUDING FLUOROSCOPY, IMAGE DOCUMENTATION AND REPORT (INCLUDES ACCESS OF SHUNT, INJECTION[S] OF CONTRAST, AND ALL NECESSARY IMAGING FROM THE ARTERIAL ANASTOMOSIS AND ADJACENT ARTERY THROUGH ENTIRE VENOUS OUTFLOW INCLUDING THE INFERIOR OR SUPERIOR VENA CAVA)
36223	PLACE CATH CAROTID/INOM ART	SELECTIVE CATHETER PLACEMENT, COMMON CAROTID OR INNOMINATE ARTERY, UNILATERAL, ANY APPROACH, WITH ANGIOGRAPHY OF THE IPSILATERAL INTRACRANIAL CAROTID CIRCULATION AND ALL ASSOCIATED RADIOLOGICAL SUPERVISION AND INTERPRETATION, INCLUDES ANGIOGRAPHY OF THE EXTRACRANIAL CAROTID AND CERVICOCEREBRAL ARCH, WHEN PERFORMED
36224	PLACE CATH CAROTD ART	SELECTIVE CATHETER PLACEMENT, INTERNAL CAROTID ARTERY, UNILATERAL, WITH ANGIOGRAPHY OF THE IPSILATERAL INTRACRANIAL CAROTID CIRCULATION AND ALL ASSOCIATED RADIOLOGICAL SUPERVISION AND INTERPRETATION, INCLUDES ANGIOGRAPHY OF THE EXTRACRANIAL CAROTID AND CERVICOCEREBRAL ARCH, WHEN PERFORMED
36225	PLACE CATH SUBCLAVIAN ART	SELECTIVE CATHETER PLACEMENT, SUBCLAVIAN OR INNOMINATE ARTERY, UNILATERAL, WITH ANGIOGRAPHY OF THE IPSILATERAL VERTEBRAL CIRCULATION AND ALL ASSOCIATED RADIOLOGICAL SUPERVISION AND INTERPRETATION, INCLUDES ANGIOGRAPHY OF T HE CERVICOCEREBRAL ARCH, WHEN PERFORMED
36226	PLACE CATH VERTEBRAL ART	SELECTIVE CATHETER PLACEMENT, VERTEBRAL ARTERY, UNILATERAL, WITH ANGIOGRAPHY OF THE IPSILATERAL VERTEBRAL CIRCULATION AND ALL ASSOCIATED RADIOLOGICAL SUPERVISION AND INTERPRETATION, INCLUDES ANGIOGRAPHY OF THE CERVICOCEREBRAL ARCH, WHEN PERFORMED
36227	PLACE CATH XTRNL CAROTID	SELECTIVE CATHETER PLACEMENT, EXTERNAL CAROTID ARTERY, UNILATERAL, WITH ANGIOGRAPHY OF THE IPSILATERAL EXTERNAL CAROTID CIRCULATION AND ALL ASSOCIATED RADIOLOGICAL SUPERVISION AND INTERPRETATION (LIST SEPARATELY IN ADDITION TO CODE FOR PRIMARY PROCEDURE)
36245	INS CATH ABD/L-EXT ART 1ST	SELECTIVE CATHETER PLACEMENT, ARTERIAL SYSTEM; EACH FIRST ORDER ABDOMINAL, PELVIC, OR LOWER EXTREMITY ARTERY BRANCH, WITHIN A VASCULAR FAMILY
36556	INSERT NON-TUNNEL CV CATH	INSERTION OF NON-TUNNELED CENTRALLY INSERTED CENTRAL VENOUS CATHETER; AGE 5 YEARS OR OLDER
36558	INSERT TUNNELED CV CATH	INSERTION OF TUNNELED CENTRALLY INSERTED CENTRAL VENOUS CATHETER, WITHOUT SUBCUTANEOUS PORT OR PUMP; AGE 5 YEARS OR OLDER
36561	INSERT TUNNELED CV CATH	INSERTION OF TUNNELED CENTRALLY INSERTED CENTRAL VENOUS ACCESS DEVICE, WITH SUBCUTANEOUS PORT; AGE 5 YEARS OR OLDER
36571	INSERT PICVAD CATH	INSERTION OF PERIPHERALLY INSERTED CENTRAL VENOUS ACCESS DEVICE, WITH SUBCUTANEOUS PORT; AGE 5 YEARS OR OLDER
36589	REMOVAL TUNNELED CV CATH	REMOVAL OF TUNNELED CENTRAL VENOUS CATHETER, WITHOUT SUBCUTANEOUS PORT OR PUMP

© 2021 Mudumbai SC et al. *JAMA Network Open.*

36901	INTRO CATH DIALYSIS CIRCUIT	INTRODUCTION OF NEEDLE(S) AND/OR CATHETER(S), DIALYSIS CIRCUIT, WITH DIAGNOSTIC ANGIOGRAPHY OF THE DIALYSIS CIRCUIT, INCLUDING ALL DIRECT PUNCTURE(S) AND CATHETER PLACEMENT(S), INJECTION(S) OF CONTRAST, ALL NECESSARY IMAGING FROM THE ARTERIAL ANASTOMOSIS AND ADJACENT ARTERY THROUGH ENTIRE VENOUS OUTFLOW INCLUDING THE INFERIOR OR SUPERIOR VENA CAVA, FLUOROSCOPIC GUIDANCE, RADIOLOGICAL SUPERVISION AND INTERPRETATION AND IMAGE DOCUMENTATION AND REPORT;
37224	FEM/POPL REVAS W/TLA	REVASCULARIZATION, ENDOVASCULAR, OPEN OR PERCUTANEOUS, FEMORAL, POPLITEAL ARTERY(S), UNILATERAL; WITH TRANSLUMINAL ANGIOPLASTY
37607	LIGATION OF A-V FISTULA	LIGATION OR BANDING OF ANGIOACCESS ARTERIOVENOUS FISTULA
38505	NEEDLE BIOPSY LYMPH NODES	BIOPSY OR EXCISION OF LYMPH NODE(S); BY NEEDLE, SUPERFICIAL (EG, CERVICAL, INGUINAL, AXILLARY)
4052F	HEMODIALYSIS VIA AV FISTULA	HEMODIALYSIS VIA FUNCTIONING ARTERIOVENOUS (AV) FISTULA (ESRD)
4053F	HEMODIALYSIS VIA AV GRAFT	HEMODIALYSIS VIA FUNCTIONING ARTERIOVENOUS (AV) GRAFT (ESRD)
4054F	HEMODIALYSIS VIA CATHETER	HEMODIALYSIS VIA CATHETER (ESRD)
43200	ESOPHAGOSCOPY FLEXIBLE BRUSH	ESOPHAGOSCOPY, FLEXIBLE, TRANSORAL; DIAGNOSTIC, INCLUDING COLLECTION OF SPECIMEN(S) BY BRUSHING OR WASHING, WHEN PERFORMED (SEPARATE PROCEDURE)
43229	ESOPHAGOSCOPY LESION ABLATE	ESOPHAGOSCOPY, FLEXIBLE, TRANSORAL; WITH ABLATION OF TUMOR(S), POLYP(S), OR OTHER LESION(S) (INCLUDES PRE- AND POST-DILATION AND GUIDE WIRE PASSAGE, WHEN PERFORMED)
43235	EGD DIAGNOSTIC BRUSH WASH	ESOPHAGOGASTRODUODENOSCOPY, FLEXIBLE, TRANSORAL; DIAGNOSTIC, INCLUDING COLLECTION OF SPECIMEN(S) BY BRUSHING OR WASHING, WHEN PERFORMED (SEPARATE PROCEDURE)
43239	EGD BIOPSY SINGLE/MULTIPLE	ESOPHAGOGASTRODUODENOSCOPY, FLEXIBLE, TRANSORAL; WITH BIOPSY, SINGLE OR MULTIPLE
43248	EGD GUIDE WIRE INSERTION	ESOPHAGOGASTRODUODENOSCOPY, FLEXIBLE, TRANSORAL; WITH INSERTION OF GUIDE WIRE FOLLOWED BY PA SSAGE OF DILATOR(S) THROUGH ESOPHAGUS OVER GUIDE WIRE
43255	EGD CONTROL BLEEDING ANY	ESOPHAGOGASTRODUODENOSCOPY, FLEXIBLE, TRANSORAL; WITH CONTROL OF BLEEDING, ANY METHOD
43265	ERCP LITHOTRIPSY CALCULI	ENDOSCOPIC RETROGRADE CHOLANGIOPANCREATOGRAPHY (ERCP); WITH DESTRUCTION OF CALCULI, ANY METHOD (EG, MECHANICAL, ELECTROHYDRAULIC, LITHOTRIPSY)
43277	ERCP EA DUCT/AMPULLA DILATE	ENDOSCOPIC RETROGRADE CHOLANGIOPANCREATOGRAPHY (ERCP); WITH TRANS-ENDOSCOPIC BALLOON DILATION OF BILIARY/PANCREATIC DUCT(S) OR OF AMPULLA (SPHINCTEROPLASTY), INCLUDING SPHINCTEROTOMY, WHEN PERFORMED, EACH DUCT
43281	LAP PARAESOPHAG HERN REPAIR	LAPAROSCOPY, SURGICAL, REPAIR OF PARAESOPHAGEAL HERNIA, INCLUDES FUNDOPLASTY, WHEN PERFORMED; WITHOUT IMPLANTATION OF MESH
44140	PARTIAL REMOVAL OF COLON	COLECTOMY, PARTIAL; WITH ANASTOMOSIS
44950	APPENDECTOMY	APPENDECTOMY;
44955	APPENDECTOMY ADD-ON	APPENDECTOMY; WHEN DONE FOR INDICATED PURPOSE AT TIME OF OTHER MAJOR PROCEDURE (NOT AS SEPARATE PROCEDURE) (LIST SEPARATELY IN ADDITION TO CODE FOR PRIMARY PROCEDURE)
49082	ABD PARACENTESIS	ABDOMINAL PARACENTESIS (DIAGNOSTIC OR THERAPEUTIC); WITHOUT IMAGING GUIDANCE
49652	LAP VENT/ABD HERNIA REPAIR	LAPAROSCOPY, SURGICAL, REPAIR, VENTRAL, UMBILICAL, SPIGELIAN OR EPIGASTRIC HERNIA (INCLUDES MESH INSERTION, WHEN PERFORMED); REDUCIBLE
50200	RENAL BIOPSY PERQ	RENAL BIOPSY; PERCUTANEOUS, BY TROCAR OR NEEDLE
51700	IRRIGATION OF BLADDER	BLADDER IRRIGATION, SIMPLE, LAVAGE AND/OR INSTILLATION

© 2021 Mudumbai SC et al. *JAMA Network Open.*

51705	CHANGE OF BLADDER TUBE	CHANGE OF CYSTOSTOMY TUBE; SIMPLE
51720	TREATMENT OF BLADDER LESION	BLADDER INSTILLATION OF ANTICARCINOGENIC AGENT (INCLUDING RETENTION TIME)
52000	CYSTOSCOPY	CYSTOURETHROSCOPY (SEPARATE PROCEDURE)
52234	CYSTOSCOPY AND TREATMENT	CYSTOURETHROSCOPY, WITH FULGURATION (INCLUDING CRYOSURGERY OR LASER SURGERY) AND/OR RESECTION OF; SMALL BLADDER TUMOR(S) (0.5 UP TO 2.0 CM)
52332	CYSTOSCOPY AND TREATMENT	CYSTOURETHROSCOPY, WITH INSERTION OF INDWELLING URETERAL STENT (EG, GIBBONS OR DOUBLE-J TYPE)
52356	CYSTO/URETERO W/LITHOTRIPSY	CYSTOURETHROSCOPY, WITH URETEROSCOPY AND/OR PYELOSCOPY; WITH LITHOTRIPSY INCLUDING INSERTION OF INDWELLING URETERAL STENT (EG, GIBBONS OR DOUBLE-J TYPE)
52601	PROSTATECTOMY (TURP)	TRANSURETHRAL ELECTROSURGICAL RESECTION OF PROSTATE, INCLUDING CONTROL OF POSTOPERATIVE BLEEDING, COMPLETE (VASECTOMY, MEATOTOMY, CYSTOURETHROSCOPY, URETHRAL CALIBRATION AND/OR DILATION, AND INTERNAL URETHROTOMY ARE INCLUDED)
54235	PENILE INJECTION	INJECTION OF CORPORA CAVERNOSA WITH PHARMACOLOGIC AGENT(S) (EG, PAPAVERINE, PHENTOLAMINE)
62311	INJECT SPINE L/S (CD)	INJECTION(S), OF DIAGNOSTIC OR THERAPEUTIC SUBSTANCE(S) (INCLUDING ANESTHETIC, ANTISPASMODIC, OPIOID, STEROID, OTHER SOLUTION), NOT INCLUDING NEUROLYTIC SUBSTANCES, INCLUDING NEEDLE OR CATHETER PLACEMENT, INCLUDES CONTRAST FOR LOCALIZATION WHEN PERFORMED, EPIDURAL OR SUBARACHNOID; LUMBAR OR SACRAL (CAUDAL)
62323	NJX INTERLAMINAR LMBR/SAC	INJECTION(S), OF DIAGNOSTIC OR THERAPEUTIC SUBSTANCE(S) (EG, ANESTHETIC, ANTISPASMODIC, OPIOID, STEROID, OTHER SOLUTION), NOT INCLUDING NEUROLYTIC SUBSTANCES, INCLUDING NEEDLE OR CATHETER PLACEMENT, INTERLAMINAR EPIDURAL OR SUBARACHNOID, LUMBAR OR SACRAL (CAUDAL); WITH IMAGING GUIDANCE (IE, FLUOROSCOPY OR CT)
66179	AQUEOUS SHUNT EYE W/O GRAFT	AQUEOUS SHUNT TO EXTRAOCULAR EQUATORIAL PLATE RESERVOIR, EXTERNAL APPROACH; WITHOUT GRAFT
66840	REMOVAL OF LENS MATERIAL	REMOVAL OF LENS MATERIAL; ASPIRATION TECHNIQUE, 1 OR MORE STAGES
66850	REMOVAL OF LENS MATERIAL	REMOVAL OF LENS MATERIAL; PHACOFRAGMENTATION TECHNIQUE (MECHANICAL OR ULTRASONIC) (EG, PHACOEMULSIFICATION), WITH ASPIRATION
66852	REMOVAL OF LENS MATERIAL	REMOVAL OF LENS MATERIAL; PARS PLANA APPROACH, WITH OR WITHOUT VITRECTOMY
66986	EXCHANGE LENS PROSTHESIS	EXCHANGE OF INTRAOCULAR LENS
66999	EYE SURGERY PROCEDURE	UNLISTED PROCEDURE, ANTERIOR SEGMENT OF EYE
67028	INJECTION EYE DRUG	INTRAVITREAL INJECTION OF A PHARMACOLOGIC AGENT (SEPARATE PROCEDURE)
67042	VIT FOR MACULAR HOLE	VITRECTOMY, MECHANICAL, PARS PLANA APPROACH; WITH REMOVAL OF INTERNAL LIMITING MEMBRANE OF RETINA (EG, FOR REPAIR OF MACULAR HOLE, DIABETIC MACULAR EDEMA), INCLUDES, IF PERFORMED, INTRAOCULAR TAMPONADE (IE, AIR, GAS OR SILICONE OIL)
67108	REPAIR DETACHED RETINA	REPAIR OF RETINAL DETACHMENT; WITH VITRECTOMY, ANY METHOD, INCLUDING, WHEN PERFORMED, AIR OR GAS TAMPONADE, FOCAL ENDOLASER PHOTOCOAGULATION, CRYOTHERAPY, DRAINAGE OF SUBRETINAL FLUID, SCLERAL BUCKLING, AND/OR REMOVAL OF LENS BY SAME TECHNIQUE
67113	REPAIR RETINAL DETACH CPLX	REPAIR OF COMPLEX RETINAL DETACHMENT (EG, PROLIFERATIVE VITREORETINOPATHY, STAGE C-1 OR GREATER, DIABETIC TRACTION RETINAL DETACHMENT, RETINOPATHY OF PREMATURITY, RETINAL TEAR OF GREATER THAN 90 DEGREES), WITH VITRECTOMY AND MEMBRANE PEELING, INCLUDING, WHEN PERFORMED, AIR, GAS, OR SILICONE OIL TAMPONADE, CRYOTHERAPY, ENDOLASER PHOTOCOAGULATION, DRAINAGE OF SUBRETINAL FLUID, SCLERAL BUCKLING, AND/OR REMOVAL OF LENS
67145	TREATMENT OF RETINA	PROPHYLAXIS OF RETINAL DETACHMENT (EG, RETINAL BREAK, LATTICE DEGENERATION) WITHOUT DRAINAGE, 1 OR MORE SESSIONS; PHOTOCOAGULATION (LASER OR XENON ARC)

© 2021 Mudumbai SC et al. *JAMA Network Open.*

67210	TREATMENT OF RETINAL LESION	DESTRUCTION OF LOCALIZED LESION OF RETINA (EG, MACULAR EDEMA, TUMORS), 1 OR MORE SESSIONS; PHOTOCOAGULATION
67228	TREATMENT X10SV RETINOPATHY	TREATMENT OF EXTENSIVE OR PROGRESSIVE RETINOPATHY (EG, DIABETIC RETINOPATHY), PHOTOCOAGULATION
68200	TREAT EYELID BY INJECTION	SUBCONJUNCTIVAL INJECTION
68761	CLOSE TEAR DUCT OPENING	CLOSURE OF THE LACRIMAL PUNCTUM; BY PLUG, EACH
69210	REMOVE IMPACTED EAR WAX UNI	REMOVAL IMPACTED CERUMEN REQUIRING INSTRUMENTATION, UNILATERAL
69220	CLEAN OUT MASTOID CAVITY	DEBRIDEMENT, MASTOIDECTOMY CAVITY, SIMPLE (EG, ROUTINE CLEANING)

© 2021 Mudumbai SC et al. *JAMA Network Open.*

eTable 2. Bivariate Analysis Describing the Association Between Patient, Surgery and Facility Characteristics, and Receipt of Any Preoperative Test Before Cataract Surgery in Fiscal Year 2017. All variables were eligible to be included in model at p<0.25.

**Patient characteristics**	**Odds ratios**	**P-value**
**Facility surgical complexity**		
Ambulatory advanced	Ref.	
Ambulatory basic	0.52	0.35
Inpatient standard	0.11	<0.00
Inpatient intermediate	0.41	0.11
Inpatient complex	0.45	0.11
Unknown	0.45	0.28
Facilities annual Cataract surgery volume	1.00	0.23
Age (per 1 years)	1.00	0.15
Female (vs. male)	1.08	0.11
**Race/Ethnicity**		
Non-Hispanic white	Ref.	
Asian	0.93	<0.00
Black or African American	1.16	<0.00
Hawaiian	0.88	<0.00
American Indian	1.02	<0.00
**Marital Status**		
Married	Ref.	
Single	0.99	0.90
Divorced	0.96	<0.01
Widowed	0.96	0.19
Never Married	1.00	0.19
**Body Mass Index**		
Normal (healthy weight)	Ref.	
Low	0.77	0.98
Obese	0.04	1.05
Overweight	0.61	1.01
**Elixhauser Index (Individual Comorbidities)**		
LX_Congestive_Heart_Failure	1.43	<0.00
ELX_Caridiac_Arrhythmia	1.38	<0.00
ELX_Pulmonary_Circulation_Disorders	1.53	<0.00
ELX_Peripheral_Vascular_Disorders	1.21	<0.00
ELX_Hypertension_Uncomplicated	1.18	<0.00
ELX_Hypertension_Complicated	1.35	<0.00
ELX_Paralysis	1.38	<0.00
ELX_GRP_Other_Neurological_Disorders	1.21	<0.00
ELX_Chronic_Pulmonary_Disease	1.21	<0.00
ELX_Diabetes_Uncomplicated	1.16	<0.00
ELX_Diabetes_Complicated	1.21	<0.00
ELX_Hypothyroidism	1.10	<0.00
ELX_Renal_Failure	1.41	<0.00
ELX_Liver_Disease	1.29	<0.00
ELX_Peptic_Ulcer_Disease_excluding_bleeding	1.29	<0.00
ELX_AIDS_HIV	1.23	<0.00
ELX_Lymphoma	2.17	<0.00
ELX_Metastatic_Cancer	2.00	<0.00
ELX_Solid_Tumor_Without_Metastasis	1.23	<0.00
ELX_Rheumatoid_Arthritis_collagen	1.21	<0.00

© 2021 Mudumbai SC et al. *JAMA Network Open.*

ELX_Coagulopathy	1.53	<0.00
ELX_Obesity	1.12	<0.00
ELX_Weight_Loss	1.39	<0.00
ELX_Fluid_Electrolyte_Disorders	1.45	<0.00
ELX_Blood_Loss_Anemia	1.64	<0.00
ELX_Alcohol_Abuse	1.14	<0.00
ELX_Drug_Abuse	1.27	<0.00
ELX_Psychoses	1.11	<0.00
ELX_Depression	1.12	<0.00
**Surgery characteristics**		
General Anesthesia	Ref.	
Monitored Anesthesia Care (MAC)	0.76	<0.00

© 2021 Mudumbai SC et al. *JAMA Network Open.*

eTable 3. Costs Per CPT Code for Laboratory Tests
CPT Code= Current Procedural Terminology. Total Costs are in dollars and include physician charges and all other charges (i.e., facility charges).

CPT Code	Total Cost ($)
71010	94.27
71015	101.61
71020	101.61
71021	111.13
71022	121.42
71023	146.79
71030	121.41
71034	225.64
71035	105.00
78451	1602.51
78452	1756.47
78453	1545.94
78454	1706.75
78466	588.34
78468	689.47
78469	1458.45
78472	637.61
78473	725.31
78481	676.61
78483	774.14
78494	642.62
78496	46.52
80047	11.60
80048	11.60
80053	14.49
81000	4.35
81001	4.35
81002	3.50
81003	3.08
81005	2.97
81007	3.52
81015	4.18
81020	5.06
82803	26.54
82805	38.92
82810	11.97
85025	10.66
85027	8.87
85345	5.91
85347	5.84
85348	5.11
93000	17.59
93005	8.86
93010	8.77
93015	79.19
93016	22.93
93017	40.90
93018	15.29
93040	13.23
93041	5.91
93042	7.28
93303	772.90
93304	660.02
93306	764.09
93307	642.33

© 2021 Mudumbai SC et al. *JAMA Network Open.*

93308	387.67
93320	56.08
93321	28.32
93325	26.39
93350	784.43
93351	831.04
93352	35.31
94010	37.17
94060	63.27
94070	62.31
94200	26.88
94250	27.61
94375	41.13
94620	58.22
94621	168.79
94726	54.83
94727	43.75
94729	56.70
94760	3.32
94761	4.81
95070	32.14
95071	38.05

© 2021 Mudumbai SC et al. *JAMA Network Open.*

eTable 4. Sensitivity Analysis: Mixed-Effects Logistic Regression Model for First Eye Surgery Describing the Association Between Patient, Surgery and Facility Characteristics, and Receipt of Any Preoperative Test Before Cataract Surgery in Fiscal Year 2017. Model was a surgery level analysis (n=31,444).

**Patient characteristics**	**Odds ratios**	**Lower Bound (95% CI)**	**Upper Bound (95%CI)**	**P-value**
**Facility surgical complexity**				
Ambulatory advanced	1.00			
Ambulatory basic	0.30	0.07	1.35	0.12
Inpatient complex	0.29	0.08	0.96	0.04
Inpatient intermediate	0.32	0.09	1.17	1.17
Inpatient standard	0.06	0.01	0.28	0.00
Unknown	0.28	0.05	1.46	0.13
Facilities annual Cataract surgery volume	0.99	0.99	1.00	0.23
Age (per 10 years)	1.00	0.99	1.00	0.76
Female (vs. male)	1.03	0.91	1.16	0.63
**Race/Ethnicity**				
Non-Hispanic white	1.00			
Asian	0.92	0.69	1.22	0.58
Black or African American	1.09	1.02	1.17	0.01
Hawaiian	0.79	0.62	1.01	0.06
American Indian	1.15	0.90	1.46	0.25
**Marital Status**				
Married	1.00			
Single	1.10	0.96	1.25	0.14
Divorced	0.99	0.87	1.14	0.96
Widowed	0.99	0.85	1.16	0.97
Never	1.06	0.92	1.24	0.38
**Body Mass Index**				
Normal (healthy weight) (reference)	1.00			
Low	0.86	0.70	1.06	0.17
Obese	0.97	0.91	1.04	0.47
Overweight	1.01	0.94	1.07	0.72
**Elixhauser Index (Number of comorbidities**	1.08	1.06	1.08	0.00
**Surgery characteristics**				
General Anesthesia	1.00			
Monitored Anesthesia Care (MAC)	0.63	0.53	0.76	0.00

© 2021 Mudumbai SC et al. *JAMA Network Open.*

eTable 5. Mixed-Effects Logistic Regression Model Describing the Association Between Patient, Surgery and Facility Characteristics, and Receipt of Preoperative Test Bundle Before Cataract Surgery in Fiscal Year 2017. The Intraclass correlation coefficients (ICCs) were .98 ($p < 0$) at the VHA facility level, .27 ($p < 0$) at the surgeon, and .05 ($p=0.05$) at the patient level.

**Patient characteristics**	**Odds ratios**	**Lower Bound (95% CI)**	**Upper Bound (95%CI)**	**P-value**
**Facility surgical complexity**				
Ambulatory advanced	1.000			
Ambulatory basic	6.21	6.21	>20.00	0.86
Inpatient complex	5.35	0.00	>20.00	0.85
Inpatient intermediate	3.20	0.00	>20.00	0.90
Inpatient standard	0.00	0.00	0.00	0.00
Unknown	0.27	0.00	>20.00	0.90
Facilities annual Cataract surgery volume	0.99	0.98	1.01	0.93
Age (per 10 years)	1.01	1.00	1.01	0.01
Female (vs. male)	0.99	0.84	1.17	0.98
**Race/Ethnicity**				
Non-Hispanic white	1.000			
Asian	0.88	0.56	1.38	0.58
Black or African American	1.06	0.97	1.16	0.15
Hawaiian	0.72	0.49	1.06	0.10
American Indian	0.82	0.59	1.15	0.26
**Marital Status**				
Married	1.000			
Single	0.87	0.73	1.03	0.11
Divorced	0.30	0.77	1.08	0.83
Widowed	0.81	0.66	0.98	0.03
Never	0.92	0.76	1.12	0.43
**Body Mass Index**				
Normal (healthy weight)	1.00			
Low	0.85	0.66	1.11	0.24
Obese	0.97	0.89	1.05	0.48
Overweight	0.96	0.88	1.04	0.40
**Elixhauser Index Number of Comorbidities**	1.03	1.02	1.05	0.00
**Surgery characteristics**				
General Anesthesia	1.00			
Monitored Anesthesia Care	0.64	0.53	0.79	0.00

© 2021 Mudumbai SC et al. *JAMA Network Open.*

eTable 6. Mixed-Effects Poisson Regression Model Describing the Association Between Patient, Surgery and Facility Characteristics, and Number of Preoperative Tests Before Cataract Surgery in Fiscal Year 2017. The intraclass correlation coefficients (ICC) were .70 (p < 0) at the VHA facility level, .42 (p < 0) at the surgeon, and .32 (p=0) at the patient level.

Patient characteristics	Point Estimate	Lower Bound (95% CI)	Upper Bound (95%CI)	P-value
**Facility surgical complexity**				
Ambulatory advanced	1.00			
Ambulatory basic	0.74	0.40	1.34	0.43
Inpatient complex	0.68	0.37	1.27	0.24
Inpatient intermediate	0.68	0.35	1.32	0.26
Inpatient standard	0.27	0.12	0.60	0.00
Unknown	0.61	0.26	1.43	0.26
Facilities annual Cataract surgery volume	1.00	1.00	1.00	0.12
Age (per 10 years)	1.00	1.00	1.00	0.52
Female (vs. male)	1.04	0.98	1.10	0.20
**Race/Ethnicity**				
Non-Hispanic white	1.000			
Asian	0.95	0.82	1.10	0.48
Black or African American	1.03	1.00	1.07	0.03
Hawaiian	0.90	0.80	1.01	0.09
American Indian	1.02	0.91	1.14	0.74
**Marital Status**				
Married	1.00			
Single	0.99	0.93	1.05	0.72
Divorced	0.97	0.93	1.05	0.40
Widowed	0.96	0.90	1.03	0.24
**Body Mass Index**				
, Normal (healthy weight)	1.00			
Low	0.92	0.84	1.01	0.09
Obese	0.98	0.96	1.01	0.25
Overweight	1.00	0.97	1.03	0.88
**Elixhauser Index Number of Comorbidities**	1.05	1.05	1.05	0.00
**Surgery characteristics**				
General Anesthesia	1.00			
Monitored Anesthesia Care (MAC)	0.82	0.66	0.99	0.00

© 2021 Mudumbai SC et al. *JAMA Network Open.*
